# Health reference intervals and values for common bottlenose dolphins (*Tursiops truncatus*), Indo-Pacific bottlenose dolphins (*Tursiops aduncus*), Pacific white-sided dolphins *(Lagenorhynchus obliquidens*), and beluga whales *(Delphinapterus leucas)*

**DOI:** 10.1371/journal.pone.0250332

**Published:** 2021-08-30

**Authors:** Lisa K. Lauderdale, Michael T. Walsh, Kevin A. Mitchell, Douglas A. Granger, Jill D. Mellen, Lance J. Miller

**Affiliations:** 1 Conservation Science and Animal Welfare Research, Chicago Zoological Society–Brookfield Zoo, Brookfield, IL, United States of America; 2 Department of Comparative, Diagnostic & Population Medicine, College of Veterinary Medicine, University of Florida, Gainesville, FL, United States of America; 3 Institute for Interdisciplinary Salivary Bioscience Research, University of California, Irvine, CA, United States of America; 4 Biology Department, Portland State University, Portland, OR, United States of America; Universita di Bologna, ITALY

## Abstract

This study reports comprehensive clinical pathology data for hematology, serum, and plasma biochemistry reference intervals for 174 apparently healthy common bottlenose dolphins (*Tursiops truncatus*) and reference values for 27 Indo-Pacific bottlenose dolphins (*Tursiops aduncus*), 13 beluga whales (*Delphinapterus leucas*), and 6 Pacific white-sided dolphins (*Lagenorhynchus obliquidens*) in zoos and aquariums accredited by the Alliance for Marine Mammal Parks and Aquariums and the Association of Zoos & Aquariums. Blood samples were collected as part of a larger study titled “Towards understanding the welfare of cetaceans in zoos and aquariums” (colloquially called the Cetacean Welfare Study). Two blood samples were collected following a standardized protocol, and two veterinarian examinations were conducted approximately six months apart between July to November 2018 and January to April 2019. Least square means, standard deviations, and 95% confidence intervals were calculated for hematology, serum, and plasma biochemical variables. Comparisons by age, gender, and month revealed statistically significant differences (*p* < 0.01) for several variables. Reference intervals and values were generated for samples tested at two laboratories for up to 56 hematologic, serum, and plasma biochemical variables. To apply these data, ZooPhysioTrak, an iOS mobile software application, was developed to provide a new resource for cetacean management. ZooPhysioTrak provides species-specific reference intervals and values based on user inputs of individual demographic and sample information. These data provide a baseline from which to compare hematological, serum, and plasma biochemical values in cetaceans in zoos and aquariums.

## Introduction

Accurate hematological, serum, and plasma biochemical reference intervals and values developed from healthy cetaceans facilitate the determination of the health status of individuals [1, 2]. Comparing the results of diagnostic laboratory tests to those of healthy individuals aids in the evaluation of a dolphin’s health condition and the establishment of treatment plans [2, 3]. Previous publications have reported clinicopathologic reference intervals or values for some of the most common cetacean species in zoos and aquariums including common bottlenose dolphins (*Tursiops truncatus*), Indo-Pacific bottlenose dolphins (*Tursiops aduncus*), beluga whales (*Delphinapterus leucas*), and Pacific white-sided dolphins (*Lagenorhynchus obliquidens*). Of these species, common bottlenose dolphins have been the focus of the most rigorous research in order to characterize hematological and serum reference intervals [4–7]. Venn-Watson and colleagues [7] provided age and sex specific reference intervals that were generated from archival blood samples from a large number of dolphins housed in open ocean pens at a single location. However, management practices and diet are known to affect several hematologic variables [8, 9] and therefore may not represent the breadth of variation in commonly used reference variables for healthy dolphins in accredited zoos and aquariums around the world.

Sparse reports of hematological, serum, or plasma values exist for Indo-Pacific bottlenose dolphins [10, 11] and Pacific white-sided dolphins [4–6] under professional care. Investigations of Indo-Pacific bottlenose dolphins focused on variations in seasonal and diurnal serum cortisol as opposed to a full range of diagnostic variables [10, 11]. Reports regarding Pacific white-sided dolphins provide hematological and plasma biochemistry values [4–6]. However, these reports are decades old and the level of health care provided to cetaceans through advanced medical protocols as well as the technology level of the equipment used has improved over time [12].

Several studies have reported on the variation in hematological, serum, and plasma biochemical values for beluga whales in zoos and aquariums [4, 8, 9, 13–16]. Baseline hematological and serum biochemical values from belugas housed in the United States [8] and hematological and plasma biochemical values from Taiwan [9] have previously been reported. Variation in their sample collection methods and tested variables emphasize the importance standardized sample collection protocols and utilizing a single lab for analysis in order to establish comparability across facilities. Blood samples are commonly analyzed at internal or commercial laboratories which results in variation in their methods and reference intervals. Further, few zoos or aquariums care for enough cetaceans within their facility to develop functional reference intervals given high inter-individual variability in physiological biomarkers in cetaceans.

Results of studies of cetaceans in zoos and aquariums are particularly valuable because blood samples can be obtained voluntarily via a trained fluke present and blood draw behavior and the animal’s health history is known. The objective of this study was to obtain samples from a large cross-section of the zoo and aquarium populations in order to derive robust reference intervals and values for more variables than previously described. To assist in overcoming importation and exportation restrictions, reference intervals were developed for samples sent to laboratories in the United States and Mexico for common bottlenose dolphins. We aimed to report values for the first time or expand the available hematological, serum, and plasma biochemical reference values for Indo-Pacific bottlenose dolphins, beluga whales, and Pacific white-sided dolphins.

## Materials and methods

### Ethics statement

This study was authorized by the management at each participating zoo and aquarium and, where applicable, was reviewed and approved by research committees. In addition, the study protocol was reviewed and approved by the U.S. Navy Marine Mammal Program Institutional Animal Care and Use Committee #123–2017.

### Subjects and facilities

Zoos and aquariums that were accredited in 2017 by the Alliance for Marine Mammal Parks and Aquariums and the Association of Zoos & Aquariums were eligible for participation in this study provided they cared for common bottlenose dolphins, Indo-Pacific bottlenose dolphins, Pacific white-sided dolphins, or beluga whales.

To select cetaceans to participate in the study, each location provided a list of demographic information of all available participants. For common bottlenose dolphins, stratified sampling was used to select participants by dividing potential subjects into groups by sex and age class (calf, juvenile, sub-adult, adult, senior, and geriatric). Age class by sex was weighted by their frequency in the group of available participants to generate a target number of participants that represented the demographic distribution across facilities. Then, participants were randomly selected within their respective category (*e*.*g*., adult female). For Indo-Pacific bottlenose dolphins, beluga whales, and Pacific white-sided dolphins, all available individuals trained to voluntarily provide biological samples were selected. Blood sample collection attempts were conducted with each dolphin approximately six months apart between July through November 2018 and January through April 2019. Time periods were selected to correspond with one peak visitor month and one non-peak visitor month at each facility.

A total of 43 facilities participated in the study by providing data from cetaceans who met the inclusion criteria. Blood samples were collected from 174 common bottlenose dolphins, 27 Indo-Pacific bottlenose dolphins, 13 beluga whales, and 6 Pacific white-sided dolphins. The Cornell University Animal Health Diagnostic Center (AHDC) laboratory analyzed 122 common bottlenose dolphins, 47 Indo-Pacific bottlenose dolphins, 21 beluga whale, and 6 Pacific white-sided dolphin samples originating from Bermuda, Hong Kong, Jamaica, Singapore, Spain, and the United States for 56 hematologic, serum, and plasma biochemical variables. The Universidad Nacional Autónoma de México (UNAM) laboratory analyzed 162 common bottlenose dolphin samples originating from Mexico for 55 hematologic and serum biochemical variables. Samples analyzed at different laboratories were not compared. As blood samples were collected through a voluntary fluke-presenting behavior (*i*.*e*., a dolphin presents its tail flukes in front of an animal care staff member and allows a blood sample to be collected), samples were not collected if the dolphin declined to comply with the requested behavior on the sample collection date. In addition, blood samples were not analyzed if they did not meet the volume required for testing. Of the samples processed at the UNAM, 94.4% were obtained from dolphins living in professionally managed ocean habitats (*i*.*e*., cordoned off sections of coastal ocean, bays, lagoons, or waterways). Of the samples process at the AHDC, 94.9% were obtained from dolphins living in professionally managed zoo/aquarium habitats (*i*.*e*., fabricated habitats with or without exposure to weather patterns).

### Sample collection

Blood samples from fasted animals were collected during the first training session of the day from the ventral or dorsal vasculature of the fluke using a voluntary tail fluke present. Flukes were cleaned with isopropyl alcohol and iodine gauze. Blood was drawn into a 6 mL BD EDTA (K2) vacutainer, a 5 mL BD vacutainer with a serum separator, and a 2.7 mL BD sodium citrate vacutainer using a 21 G, 3/4” infusion set. Two blood smears were made from the EDTA vacutainer within one hour of collection. Slides were not fixed or stained. After collection, the serum tubes sat at room temperature for 45 minutes until clot formation. The tube was centrifuged at 112 x g for 5 minutes. The serum was aspirated using a pipette and transferred to a 4 mL cryovial. Plasma vacutainers were immediately centrifuged at 112 x g. Plasma was aspirated using a pipette and transferred to a 1.2 mL cryovial. All samples were stored in a refrigerator at or below 4° C until shipping the same day as collection. Blood samples were tested in real time as they were collected and were not stored frozen for batch analysis.

Samples originating in Mexico were shipped refrigerated to the UNAM for analysis. Hematology testing was performed by hematology analyzer EXIGO Vet® and blood chemistry was performed by biochemistry analyzer Dirui CS-T240 model. Samples originating from all other countries were shipped to the AHDC for analysis. Hematology testing was performed by hematology analyzer ADVIA 2120 and blood chemistry was performed by automated wet chemistry analyzer Cobas C501. The fibrinogen assay (Clauss method) was performed using an automated clot detection instrument (STA Compact, Diagnostica Stago, Parsippany NJ) with the manufacturer’s reagents, standards, and quality control materials (Fibrinogen, Diagnostica Stago; System control N & P, Diagnostica Stago). Hematological, serum, and plasma biochemical values included in the study were limited to those reported by each laboratory.

Veterinary examinations, cytology findings, and blood reports for all study participants were blinded and then evaluated by external veterinarians (*i*.*e*., veterinarians not at the participating facilities) with expertise in marine mammals. Variables included in the analysis were sex, age at the time of sampling, and month the sample was collected. The list of hematologic, serum biochemical, and plasma variables tested at AHDC and UNAM are listed in S1 and S2 Tables.

### Statistical analysis

In order to create reference intervals and values for blood variables in healthy cetaceans, only samples from healthy whales and dolphins were included in the analyses. The expert veterinarians and pathologists reviewed the blinded results of full physical examinations and cytology findings (from chuff, gastric, and fecal samples) to determine the health status of each individual. A dolphin was considered to be healthy if they had no known disease, had an absence of abnormal clinical signs, were not on any medications that could potentially affect blood values, and did not receive antimicrobial treatments within 14 days before or after sampling. Whales and dolphins that were administered eye drops, nutritional supplements, or contraceptives were maintained in the study. Pregnant females were excluded from the final analysis.

Analyses were completed using the software package SPSS 21 and Microsoft Excel. For common bottlenose dolphins, Indo-Pacific bottlenose dolphins, and beluga whales, mean clinicopathologic values were compared by sex, age, age^2^, and month (controlling for facility ID and animal ID) by use of a mixed model to control for repeated samples. Age^2^ was included in the model to account for non-linear relationships between age and the dependent variables. Samples from common bottlenose dolphins tested at AHDC and UNAM were analyzed separately and not compared. Significance was defined as *p* < 0.01. A full mixed model using a training set that included 70% of the data set was created. The data were randomly split by observation rather than by individual to include a variety of individuals from multiple locations, a range of ages, and sample months. If significant differences were identified in variables by sex, age, age^2^, or month, models were run with only significant variables and reference intervals or values were developed using the appropriate parameters. For example, if sex and month were significant predictors, then they were included as parameters in the reference interval computations.

Prediction equations were extracted from each 70% training model and tested on the remaining testing subset (30%) of data. These predicted values were compared to the actual values by assessing the output from the mixed models, means, and standard deviations of the predicted value relative to the observed value.

Reduced linear models were produced that excluded both animal ID and facility ID for application to future samples. If significant differences were identified in variables by sex, age, age^2^, or month, models were run with only significant variables and reference intervals or values were developed using the appropriate parameters. If no significant predictors were identified, reference intervals and values were established from values that lay within two standard deviations from the mean for common bottlenose dolphins, Indo-Pacific bottlenose dolphins, and beluga whales. Reference intervals were produced for common bottlenose dolphins. As the sample size was smaller than 40 individuals, reference values were produced for Indo-Pacific bottlenose dolphins, Pacific white-sided dolphins, and beluga whales.

For variables with significant predictors, reference intervals or values (*i*.*e*., 95% confidence intervals) were developed using the following function:

$$95\%\;\text{CI}=\hat{Y}+/-(\left({\text{t}}_{\text{crit}}\right)({\tilde{sd}}_{Y_{o}-\hat{Y_{o}}}))$$

where $\hat{Y}$ = predicted value, t_crit_ = critical value, and ${\tilde{sd}}_{Y_{o}-\hat{Y_{o}}}$ = standard error of the estimate for a single observation. The standard error of the estimate for a single observation with one predictor was calculated as:

$${\tilde{sd}}_{Y_{o}-\hat{Y_{o}}}={\tilde{sd}}_{Y-\hat{Y}}\sqrt{1+\frac{1}{n}+\frac{{\left(X_{o}-\bar{X}\right)}^{2}}{n\;sd_{X}^{2}}}$$

where ${\tilde{sd}}_{Y-\hat{Y}}$ 0020 = standard error of the estimate, *n* = sample size, *X*_*o*_ = score on the predictor, $\bar{X}$ = mean of the predictor, *sd*_*X*_ = standard deviation of the predictor [17].

The standard error of the estimate for a single observation with multiple predictors was calculated as:

$${\tilde{sd}}_{Y_{o}-\hat{Y_{o}}}=\frac{{\tilde{sd}}_{Y-\hat{Y}}}{\sqrt{n}}\sqrt{n+1+\sum \frac{z_{io}{}^{2}}{1-R_{i}^{2}}-2\sum \frac{\beta_{ij}z_{io}z_{jo}}{1-R_{i}^{2}}},$$

where the first summation is over *k* IVs and the second summation is over *k*(*k*-1)/2 pairs of IVs expressed as standard scores, ${\tilde{sd}}_{\text{Y}-\hat{\text{Y}}}$ = standard error of the estimate, *z*_*io*_ = absolute value of the IV, $R_{i\cdot 12\ldots (i)\ldots k}^{2}=R_{i}^{2}$, and *β*_*ij*_ = standardized partial regression coefficient [17].

Due to the small sample size for Pacific white-sided dolphins, non-parametric reference values were generated. Data were ranked in order of magnitude and values between the 10th and 90th percentiles were retained as the reference values [18].

### ZooPhysioTrak

ZooPhysioTrak is a native iOS mobile application that is compatible with the iPad, iPhone, and iPod Touch. Native applications are well-integrated with the operating system and do not utilize a web server or live internet connection during operation. ZooPhysioTrak was written in the Swift programming language. Test versions of ZooPhysioTrak were distributed via TestFlight. In order to ensure accurate operation, the build process for ZooPhysioTrak included automated unit testing to confirm it was producing and displaying accurate results.

Several tests were employed based on a walkthrough of the statistical analysis with example values. They ensured that the calculation was done correctly, produced a correct numerical result, showed a correct textual display of the range, and displayed a correct unit. Within the program’s database, variables with significant predictors also contained example inputs and expected outputs; these were also tested to confirm the calculation for that variable was correct. Miscellaneous tests ensured that the program data was internally consistent, and that the ordering of displayed values was as desired.

## Results

All prediction equations from full mixed models and reduced linear models were based on models created with the training data subset (Table 1). Prediction equations and *r*^2^ for the full mixed models are given in Table 2. For common bottlenose dolphins samples analyzed at the ADHC, 12 variables were significantly affected by one or more of the predictors in the mixed models. Female dolphins were significantly more likely than males to have higher chloride values and lower hematocrit and RBC values. Values of platelet count and creatinine kinase significantly decreased with age while triglycerides and creatine significantly increased with age. Alkaline phosphatase significantly decreased with age and increased with age^2^ indicating that the effect lessoned with age. Reticulocyte count and absolute reticulocyte count were significantly lower as the calendar year progressed (*i*.*e*., winter/late rainy season and varying photoperiods dependent upon the geographic location). Values of lymphocytes were significantly higher for female dolphins and significantly decreased with age. MPV values significantly increased with age and were significantly lower early in the year. Sex, age, age^2^, and month were not significant predictors for the remaining variables.

**Table 1:** Fit of predictions on training and testing datasets from linear regression models.

Variable	Data Set	n	Equation	r^2^
**Species: *Tursiops truncatus***				
**Lab: AHDC**				
Hematocrit	Training	83	44.140 + -2.307 * Sex	0.134
	Testing	37	41.944 + 0.213 * Sex	0.002
Hemoglobin	Training	83	14.921 + -0.682 * Sex	0.960
	Testing	37	14.083 + 0.354 * Sex	0.041
RBC	Training	82	3.428 + -0.273 * Sex	0.240
	Testing	37	3.267 + -0.125 * Sex	0.037
MCH	Training	83	43.660 + 1.590 * Sex	0.081
	Testing	37	42.500 + 3.395 * Sex	0.347
Lymphocytes	Training	83	1.000 + 0.400 * Sex	0.109
	Testing	37	1.044 + 0.203 * Sex	0.043
Chloride	Training	37	116.660 + 2.740 * Sex	0.165
	Testing	38	118.053 + -1.158 * Sex	0.010
MCV	Training	37	128.702 + 4.048 * Sex	0.095
	Testing	37	126.278 + 7.985 * Sex	0.282
Platelet Count	Training	82	134.418 + -1.148 * Age	0.283
	Testing	36	131.292 + -0.756 * Age	0.145
MPV	Training	36	13.248 + 0.088 * Age	0.242
	Testing	36	13.689 + 0.044 * Age	0.061
Reticulocyte Count	Training	83	3.357 + -0.146 * Month	0.080
	Testing	34	3.781 + -0.199 * Month	0.088
Absolute Reticulocyte Count	Training	83	110.177 + -5.595 * Month	0.236
	Testing	33	113.337 + -5.405 * Month	0.077
Alkaline Phosphatase	Training	82	753.668 + -32.410 * Age + 0.502 * Age^2^	0.380
	Testing	38	811.050 + -40.480 * Age + 0.700 * Age^2^	0.549
**Species: *Tursiops truncatus***				
**Lab: UNAM**				
Alkaline Phosphatase	Training	113	619.388 + -158.935 * Sex	0.089
(Fosfatasa alcalina)	Testing	50	651.036 + -20.036 * Sex	0.001
Triglycerides	Training	113	0.598 + 0.207 * Sex	0.093
(Trigliceridos)	Testing	50	0.567 + 0.293 * Sex	0.209
Hematocrit	Training	113	40.927 + 0.156 * Month	0.067
(Hematocrito)	Testing	50	40.376 + 0.307 * Month	0.231
RDWa	Training	108	99.361 + 1.148 * Month	0.147
	Testing	49	97.469 + 1.314 * Month	0.133
Pt/Fin	Training	59	46.140 + -1.163 * Month	0.139
	Testing	27	52.630 + -1.670 * Month	0.218
Total Protein	Training	113	64.196 + .360 * Month	0.063
(Proteinas totales)	Testing	50	63.925 + 0.242 * Month	0.030
Phosphorus	Training	113	1.254 + 0.018 * Month	0.098
(Fosforo)	Testing	50	1.357 + 0.003 * Month	0.002
Ca/P Ratio	Training	113	1.960 + -0.045 * Month	0.079
(Relacion Ca/P)	Testing	50	1.728 + -0.012 * Month	0.024
Potassium	Training	113	3.487 + -0.019 * Month	0.060
(Potasio)	Testing	50	3.600 + -0.028 * Month	0.087
Bicarbonate	Training	113	25.426 + 0.370 * Month	0.136
(Bicarbonato)	Testing	50	26.392 + 0.307 * Month	0.109
Strong Ion Difference	Training	113	32.316 + -0.500 * Month	0.264
(Diferencia de iones Fuertes)	Testing	50	31.876 + -0.514 * Month	0.087
Amylase	Training	111	2.398 + 0.579 * Month	0.288
(Amilasa)	Testing	50	2.707 + 0.543 * Month	0.353
Lipase	Training	113	29.245 + -0.848 * Month	0.060
(Lipasa)	Testing	50	27.316 + -0.593 * Month	0.023
**Species: *Tursiops aduncus***				
**Lab: AHDC**				
Eosinophils	Training	33	1.190 + -0.109 * Month	0.283
	Testing	14	1.614 + -0.178 * Month	0.735
Bicarbonate	Training	33	26.215 + -0.369 * Month	0.244
	Testing	14	27.792 + -0.615 * Month	0.244
Calcium	Training	33	9.664 + -0.086 * Month	0.305
	Testing	14	9.721 + -0.089 * Month	0.171
Total Protein	Training	33	7.319 + -0.135 * Month	0.404
	Testing	14	6.525 + -0.006 * Month	< 0.001
ALT	Training	33	16.376 + 10.608 * Month	0.360
	Testing	14	56.792 + 3.760 * Month	0.077
Lipemia	Training	33	4.179 + 0.495 * Month	0.361
	Testing	14	4.417 + 0.604 * Month	0.269
MPV	Training	23	13.381 + 0.414 * Age + -0.011 * Age^2^	0.404
	Testing	7	18.212 + -0.492 * Age + 0.02 * Age^2^	0.468
Creatinine	Training	33	1.329 + 0.048 * Age + -0.001 * Age^2^	0.361
	Testing	14	1.317 + 0.049 * Age + -0.002 * Age^2^	0.324
Alkaline Phosphatase	Training	33	1597.449 + -82.298 * Age + 1.599 * Age^2^	0.441
	Testing	14	1481.393 + -111.018 * Age + 3.097 * Age^2^	0.476
TIBC	Training	33	458.211 + -14.734 * Age + 0.288 * Age^2^+ 78.360 * Sex	0.529
	Testing	14	522.599 + -18.247 * Age + 0.422 * Age^2^ + -5.064 * Sex	0.517
Lymphocytes	Training	33	2.064 + -0.092 * Age + 0.002 * Age^2^ + -0.097 * Month	0.418
	Testing	14	23.641 + 1.684 * Age + -0.049 * Age^2^ + 2.500 * Month	0.850
Urea Nitrogen	Training	33	35.065 + 1.002 * Age + -0.032 * Age^2^ + 1.410 * Month	0.565
	Testing	14	23.641 + 1.684 * Age + -0.049 * Age^2^ + 2.500 * Month	0.556
Fibrinogen	Training	33	331.394 + -5.885 * Age + 0.140 * Age^2^ + -10.344 * Month	0.512
	Testing	14	318.741 + -4.86 * Age + 0.096 * Age^2^ + -5.087 * Month	0.556
**Species: *Delphinapterus leucas***				
**Lab: AHDC**				
Albumin	Training	15	6.519 + -0.190 * Age + 0.004 * Age^2^	0.669
	Testing	6	6.499 + -0.225 * Age + 0.006 * Age^2^	0.738

*Note*. n refers to the number of samples.

Training sets included 70% of the data and testing sets included 30% of data.

**Table 2:** Model estimates for predicting blood values.

Model	Variable	Parameter	Estimate	Standard Error	*t*	p
**Species: *Tursiops truncatus***						
**Lab: AHDC**						
i	Hematocrit	Intercept	44.06	0.45	97.38	< 0.001
		Sex	-2.31	0.68	-3.42	0.001
ii	RBC	Intercept	3.41	0.04	87.86	< 0.001
		Sex	-0.26	0.06	-4.52	< 0.001
iii	Chloride	Intercept	116.63	0.43	269.58	< 0.001
		Sex	2.76	0.67	4.14	< 0.001
iv	Platelet Count	Intercept	132.46	5.36	24.72	< 0.001
		Age	-1.11	0.23	-4.85	< 0.001
v	Creatine Kinase	Intercept	162.03	8.61	18.82	< 0.001
		Age	-1.40	0.37	-3.83	< 0.001
vi	Triglycerides	Intercept	37.26	5.11	7.29	< 0.001
		Age	0.79	0.22	3.56	0.001
vii	Creatinine	Intercept	1.40	0.07	19.29	< 0.001
		Age	0.01	< 0.01	2.91	0.005
viii	Alkaline Phosphatase	Intercept	739.79	69.70	10.61	< 0.001
		Age	-31.59	6.85	-4.61	< 0.001
		Age^2^	0.49	0.14	3.49	0.001
ix	Reticulocyte Count	Intercept	3.59	0.27	13.46	< 0.001
		Month	-0.19	0.04	-5.26	< 0.001
x	Absolute Reticulocyte Count	Intercept	113.80	6.49	17.54	< 0.001
		Month	-6.12	0.98	-6.25	< 0.001
xi	Lymphocytes	Intercept	1.31	0.14	9.51	< 0.001
		Age	-0.02	0.01	-3.14	0.003
		Sex	0.44	0.13	3.43	0.001
xii	MPV	Intercept	13.89	0.50	27.89	< 0.001
		Age	0.09	0.02	4.27	< 0.001
		Month	-0.11	0.03	-3.61	0.002
**Species: *Tursiops truncatus***						
**Lab: UNAM**						
xiii	Triglycerides	Intercept	0.59	0.05	12.55	< 0.001
		Sex	0.21	0.06	3.26	0.002
xiv	Hematocrit	Intercept	40.87	0.37	110.43	< 0.001
		Month	0.16	0.05	3.49	0.001
xv	Reticulocytes	Intercept	1.86	0.13	13.96	< 0.001
		Month	-0.05	0.02	-2.70	0.008
xvi	Monocytes	Intercept	0.24	0.02	11.55	< 0.001
		Month	-0.01	< 0.01	-2.98	0.004
xvii	RDWa	Intercept	99.33	1.88	52.76	< 0.001
		Month	1.15	0.26	4.47	< 0.001
xviii	Pt/Fin	Intercept	43.69	1.59	27.49	< 0.001
		Month	-0.95	0.23	-4.13	< 0.001
xix	Total Protein	Intercept	64.26	0.79	81.85	< 0.001
		Month	0.35	0.11	3.29	0.002
xx	Globulin	Intercept	23.39	0.62	37.97	< 0.001
		Month	0.23	0.08	2.69	0.009
xxi	Phosphorus	Intercept	1.27	0.03	37.67	< 0.001
		Month	0.02	< 0.01	3.78	< 0.001
xxii	Ca/P Ratio	Intercept	1.84	0.09	21.10	< 0.001
		Month	-0.02	0.01	-2.91	0.007
xxiii	Potassium	Intercept	3.50	0.05	76.11	< 0.001
		Month	-0.02	0.01	-3.52	0.001
xxiv	Sodium	Intercept	155.07	0.66	234.42	< 0.001
		Month	-0.33	0.09	-3.71	< 0.001
xxv	Bicarbonate	Intercept	25.44	0.64	39.70	< 0.001
		Month	0.37	0.10	3.83	< 0.001
xxvi	Strong Ion Ratio	Intercept	32.32	0.55	58.59	< 0.001
		Month	-0.50	0.08	-6.38	< 0.001
xxvii	Amylase	Intercept	2.38	0.61	3.92	< 0.001
		Month	0.58	0.08	6.87	< 0.001
xxviii	Lipase	Intercept	29.73	2.11	14.07	< 0.001
		Month	-0.96	0.28	-3.41	0.001
**Species: *Tursiops aduncus***						
**Lab: AHDC**						
xxix	Cholesterol	Intercept	137.36	6.60	20.80	< 0.001
		Sex	31.56	9.92	3.18	0.004
xxx	Triglycerides	Intercept	32.69	2.40	13.61	< 0.001
		Sex	13.64	3.61	3.78	0.001
xxxi	Hematocrit	Intercept	42.74	1.35	31.66	< 0.001
		Age	0.08	0.06	1.34	0.199
xxxii	Alkaline Phosphatase	Intercept	1212.29	146.37	8.28	< 0.001
		Age	-22.58	6.45	-3.50	0.002
xxxiii	MPV	Intercept	12.06	< 0.01	22485.33	< 0.001
		Age	0.63	< 0.01	7136.66	< 0.001
		Age^2^	-0.02	< 0.01	-6080.57	< 0.001
xxxiv	Creatinine	Intercept	1.32	0.13	10.28	< 0.001
		Age	0.05	0.01	3.25	0.003
		Age^2^	< 0.01	< 0.01	-3.67	0.002
xxxv	MCHC	Intercept	34.78	< 0.01	45735233.10	< 0.001
		Age	0.12	< 0.01	1073540.84	< 0.001
		Age^2^	< 0.01	< 0.01	-952034.69	< 0.001
xxxvi	MCV	Intercept	113.91	1.09	104.34	< 0.001
		Month	0.44	0.06	7.18	0.005
xxxvii	Segmented Neutrophils	Intercept	4.04	0.53	7.69	< 0.001
		Month	-0.27	0.08	-3.43	0.002
xxxviii	Eosinophils	Intercept	1.34	0.19	6.99	< 0.001
		Month	-0.13	0.03	-4.19	< 0.001
xxxix	Bicarbonate	Intercept	26.95	0.58	46.60	< 0.001
		Month	-0.48	0.09	-5.47	< 0.001
xxxx	Calcium	Intercept	9.62	0.11	84.52	< 0.001
		Month	-0.08	0.02	-4.44	< 0.001
xxxxi	Total Protein	Intercept	7.11	0.13	53.12	< 0.001
		Month	-0.11	0.02	-5.49	< 0.001
xxxxii	Albumin	Intercept	5.06	0.11	46.98	< 0.001
		Month	-0.06	0.02	-3.78	0.001
xxxxiii	ALT	Intercept	27.16	12.52	2.17	0.042
		Month	9.03	1.89	4.77	< 0.001
xxxxiv	AST	Intercept	199.62	36.72	5.44	< 0.001
		Month	18.30	5.01	3.66	0.004
xxxxv	Lipase	Intercept	4.31	0.75	5.71	< 0.001
		Month	0.33	0.12	2.80	0.010
xxxxvi	Lipemia	Intercept	4.86	0.64	7.60	< 0.001
		Month	0.42	0.10	4.24	< 0.001
xxxxvii	TIBC	Intercept	411.81	25.36	16.24	< 0.001
		Sex	85.36	23.29	3.67	0.001
		Age	-5.30	1.21	-4.37	< 0.001
xxxxviii	Hemoglobin	Intercept	15.75	0.02	654.43	< 0.001
		Sex	1.26	0.30	4.18	< 0.001
		Age	-0.13	0.01	-9.78	< 0.001
		Age^2^	< 0.01	< 0.01	5.69	< 0.001
xxxxix	Lymphocytes	Intercept	2.08	0.28	7.33	< 0.001
		Age	-0.09	0.02	-3.82	0.001
		Age^2^	< 0.01	< 0.01	3.61	0.001
		Month	-0.10	0.03	-3.04	0.005
xxxxx	Urea Nitrogen	Intercept	35.20	3.25	10.82	< 0.001
		Age	1.03	0.31	3.35	0.003
		Age^2^	-0.03	0.01	-3.93	0.001
		Month	1.36	0.32	4.24	< 0.001
xxxxxi	Fibrinogen	Intercept	335.41	16.51	20.32	< 0.001
		Sex	23.76	7.74	3.07	0.009
		Age	-6.68	1.38	-4.83	0.001
		Age^2^	0.15	0.04	3.81	0.003
		Month	-11.08	2.17	-5.11	< 0.001
**Species: *Delphinapterus leucas***						
**Lab: AHDC**						
xxxxxii	Calcium	Intercept	11.33	0.14	83.07	< 0.001
		Age	-0.16	0.01	-12.38	< 0.001
		Age^2^	< 0.01	< 0.01	10.98	< 0.001
xxxxxiii	Reticulocyte Count	Intercept	2.15	0.26	8.39	< 0.001
		Sex	0.38	0.10	3.73	0.004
		Age	-0.13	0.03	-4.95	0.002
		Age^2^	< 0.01	< 0.01	4.58	0.001
xxxxxiv	Absolute Reticulocyte Count	Intercept	74.57	7.31	10.20	< 0.001
		Sex	10.17	2.76	3.69	0.005
		Age	-5.01	0.75	-6.68	< 0.001
		Age^2^	0.11	0.02	6.59	< 0.001

Mixed models included the explanatory variables found to be significant predictors. Possible fixed effects included: sex, age, age^2^, and month. Random effects included: animal ID and facility ID.

For common bottlenose dolphin samples analyzed at the UNAM, 16 variables were significantly affected by either month or sex predictors in the mixed models. Female dolphins were more likely than male dolphins to have higher triglyceride values. Dolphins were significantly more likely to have higher hematocrit, RDWa, total protein, globulin, phosphorus, bicarbonate, and amylase values as the calendar year progressed (*i*.*e*., winter/late rainy season). Reticulocytes, monocytes, Pt/Fin, Ca/P ratio, potassium, sodium, strong ion ratio, and lipase were significantly lower near the end of the year. There were no significant differences in all other variables.

For Indo-Pacific bottlenose dolphins samples analyzed at the ADHC, 24 variables were significantly affected by one or more of the predictors in the mixed models. Female dolphins were significantly more likely than male dolphins to have higher cholesterol and triglyceride values. Hematocrit values were significantly higher with age and alkaline phosphatase values significantly decreased with age. As age and age^2^ increased, creatinine, MPV, and MCHC values significantly increased. Late in the year, MCV, ALT, AST, lipase, and lipemia values were significantly higher when compared to early in the year. Values for segmented neutrophils, eosinophils, bicarbonate, calcium, total protein, and albumin were significantly lower early in the year when compared to late in the year. TIBC values were significantly higher for female dolphins than male dolphins and decreased with age. Values of hemoglobin were significantly higher for female dolphins than male dolphins and were significantly lower with increased age and higher on age^2^ indicating that the effect lessoned with age. Lymphocyte values decreased with age, increased with age^2^, and were significantly lower as the year progressed. Urea nitrogen values increased with age and were significantly lower as the year progressed. Values of fibrinogen were significantly higher in female dolphins when compared to male dolphins, decreased with age, and were lower as the year progressed. No significant differences were identified for all other variables.

For beluga whale samples analyzed at the ADHC, three variables were significantly affected by two or more of the predictors in the mixed models. Values of calcium were significantly lower as age increase and were higher as age^2^ increased indicating that the effect lessoned with age. Both reticulocyte count and absolute reticulocyte count were significantly higher in female dolphins than male dolphins, were lower with age and higher as age^2^ increased indicating that the effect lessoned with age. The remaining variables were not significantly predicted by sex, age, age^2^, or month.

For application to future samples, the results of the linear regression (without controlling for animal ID and facility ID) are provided in Table 3. General reference intervals and values (without accounting for sex, age, age^2^, and month) are presented in Table 4 for variables in which there were no significant predictors or if access to the iOS ZooPhysioTrak app is not available.

**Table 3:** Model estimates for predicting blood values.

Model	Variable	Parameter	Estimate	Standard Error	*t*	p
**Species: *Tursiops truncatus***						
**Lab: AHDC**						
i	Hematocrit	Intercept	44.14	0.43	102.82	< 0.001
		Sex	-2.31	0.65	-3.54	0.001
ii	Hemoglobin	Intercept	14.92	0.15	97.30	< 0.001
		Sex	-0.68	0.23	-2.93	0.004
iii	RBC	Intercept	3.43	0.04	95.30	< 0.001
		Sex	-0.27	0.05	-5.02	< 0.001
iv	MCH	Intercept	43.66	0.39	111.54	< 0.001
		Sex	1.59	0.59	2.68	0.009
v	Lymphocytes	Intercept	1.00	0.08	11.92	< 0.001
		Sex	0.40	0.13	3.14	0.002
vi	Chloride	Intercept	116.66	0.45	258.64	< 0.001
		Sex	2.74	0.69	3.97	< 0.001
vii	MCV	Intercept	128.70	0.91	140.81	< 0.001
		Sex	4.05	1.39	2.92	0.005
viii	Platelet Count	Intercept	134.42	4.78	28.12	< 0.001
		Age	-1.15	0.20	-5.62	< 0.001
ix	MPV	Intercept	13.25	0.41	32.70	< 0.001
		Age	0.09	0.02	5.06	< 0.001
x	Alkaline Phosphatase	Intercept	753.67	67.05	11.24	< 0.001
		Age	-32.41	6.65	-4.88	< 0.001
		Age^2^	0.50	0.14	3.68	< 0.001
xi	Reticulocyte Count	Intercept	3.36	0.34	9.97	< 0.001
		Month	-0.15	0.06	-2.66	0.009
xii	Absolute Reticulocyte Count	Intercept	110.18	6.88	16.01	< 0.001
		Month	-5.60	1.12	-5.00	< 0.001
**Species: *Tursiops truncatus***						
**Lab: UNAM**						
xiii	Alkaline Phosphatase	Intercept	619.39	36.41	17.01	< 0.001
		Sex	-158.94	48.38	-3.29	0.001
xiv	Triglycerides	Intercept	0.60	0.05	12.94	< 0.001
		Sex	0.21	0.06	3.37	0.001
xv	Hematocrit	Intercept	40.93	0.41	100.99	< 0.001
		Month	0.16	0.06	2.76	0.007
xvi	RDWa	Intercept	99.36	1.93	51.57	< 0.001
		Month	1.15	0.27	4.27	< 0.001
xvii	Pt/Fin	Intercept	46.14	3.64	12.68	< 0.001
		Month	-1.16	0.38	-3.03	0.004
xviii	Total Protein	Intercept	64.20	0.93	69.31	< 0.001
		Month	0.36	0.13	2.73	0.007
xix	Phosphorus	Intercept	1.25	0.04	34.17	< 0.001
		Month	0.02	0.01	3.47	0.001
xx	Ca/P Ratio	Intercept	1.96	0.10	19.04	< 0.001
		Month	-0.05	0.02	-3.08	0.003
xxi	Potassium	Intercept	3.49	0.05	69.41	< 0.001
		Month	-0.02	0.01	-2.66	0.009
xxii	Bicarbonate	Intercept	25.43	0.62	40.90	< 0.001
		Month	0.37	0.09	4.18	< 0.001
xxiii	Strong Ion Ratio	Intercept	32.32	0.56	58.04	< 0.001
		Month	-0.50	0.08	-6.31	< 0.001
xxiv	Amylase	Intercept	2.40	0.62	3.88	< 0.001
		Month	0.58	0.09	6.64	< 0.001
xxv	Lipase	Intercept	29.25	2.24	13.03	< 0.001
		Month	-0.85	0.32	-2.65	0.009
**Species: *Tursiops aduncus***						
**Lab: AHDC**						
xxvi	Eosinophils	Intercept	1.19	0.19	6.15	< 0.001
		Month	-0.11	0.03	-3.49	0.001
xxvii	Bicarbonate	Intercept	26.22	0.72	36.35	< 0.001
		Month	-0.37	0.12	-3.16	0.003
xxviii	Calcium	Intercept	9.66	0.14	67.20	< 0.001
		Month	-0.09	0.02	-3.69	0.001
xxix	Total Protein	Intercept	7.32	0.18	40.09	< 0.001
		Month	-0.14	0.03	-4.58	< 0.001
xxx	ALT	Intercept	16.38	15.71	1.04	0.305
		Month	10.61	2.54	4.17	< 0.001
xxxi	Lipemia	Intercept	4.18	0.73	5.71	< 0.001
		Month	0.50	0.12	4.18	< 0.001
xxxii	MPV	Intercept	13.38	1.14	11.77	< 0.001
		Age	0.41	0.13	3.28	0.004
		Age^2^	-0.01	< 0.01	-3.61	0.002
xxxiii	Creatinine	Intercept	1.33	0.12	10.74	< 0.001
		Age	0.05	0.01	3.37	0.002
		Age^2^	-0.01	< 0.01	-3.89	0.001
xxxiv	Alkaline Phosphatase	Intercept	1597.45	185.91	8.59	< 0.001
		Age	-82.30	21.26	-3.87	0.001
		Age^2^	1.60	0.55	2.91	0.007
xxxv	TIBC	Intercept	458.21	31.08	14.74	< 0.001
		Sex	78.36	21.55	3.64	0.001
		Age	-14.73	3.52	-4.19	< 0.001
		Age^2^	0.29	0.09	3.16	0.004
xxxvi	Lymphocytes	Intercept	2.06	0.31	6.74	< 0.001
		Age	-0.09	0.03	-3.46	0.002
		Age^2^	< 0.01	< 0.01	3.30	0.003
		Month	-0.10	0.03	-2.86	0.008
xxxvii	Urea Nitrogen	Intercept	35.07	3.48	10.08	< 0.001
		Age	1.00	0.30	3.32	0.002
		Age^2^	-0.03	0.01	-4.05	< 0.001
		Month	1.41	0.38	3.67	0.001
xxxviii	Fibrinogen	Intercept	331.39	21.28	15.57	< 0.001
		Age	-5.89	1.85	-3.19	0.003
		Age^2^	0.14	0.05	2.94	0.006
		Month	-10.34	2.35	-4.40	< 0.001
**Species: *Delphinapterus leucas***						
**Lab: AHDC**						
xxxix	Albumin	Intercept	6.52	0.43	15.26	< 0.001
		Age	-0.19	0.05	-4.26	0.001
		Age^2^	< 0.01	< 0.01	3.82	0.002

Linear regression models included the explanatory variables found to be significant predictors. Possible fixed factors included: sex, age, age^2^, and month.

**Table 4:** General reference intervals and values for blood values for healthy cetaceans.

Variable (Unit)	n	Reference Interval or Value
**Species: *Tursiops truncatus***		
**Lab: AHDC**		
Hematocrit (%)	116	37.17–47.89
Hemoglobin (g/dL)	114	12.69–16.06
RBC (mill/uL)	116	2.73–3.82
MCV (fL)	118	117.36–143.56
MCH (pg)	117	38.95–49.61
MCHC (g/dL)	120	31.81–36.10
RDW (%)	117	11.55–14.83
Reticulocyte Count (%)	113	0.08–4.88
Absolute Reticulocyte Count (thou/uL)	112	7.54–151.16
Nucleated Red Blood Cells (/100 WBC)	119	0.00–3.10
WBC (thou/uL)	113	3.13–7.68
Segmented Neutrophils (thou/uL)	114	1.45–5.24
Band Neutrophils (thou/uL)	119	0.00–0.02
Lymphocytes(thou/uL)	117	0.08–2.17
Monocytes (thou/uL)	117	0.00–0.47
Eosinophils (thou/uL)	114	0.02–1.29
Basophils (thou/uL)	120	0.00–0.07
Platelet Count (thou/uL)	113	67.00–156.91
MPV (fL)	114	10.90–18.56
TP-Ref (g/dL)	109	6.43–8.51
Sodium (mEq/L)	116	152.21–160.49
Potassium (mEq/L)	114	3.18–4.29
Chloride (mEq/L)	117	113.97–122.62
Bicarbonate (mEq/L)	115	19.37–29.45
Anion Gap (mEq/L)	116	12.03–23.21
Na/K Ratio	114	34.96–48.19
Urea Nitrogen (mg/dL)	113	36.31–59.50
Creatinine (mg/dL)	114	1.07–1.95
Calcium (mg/dL)	114	8.47–9.95
Phosphate (mg/dL)	119	3.28–5.99
Magnesium (mEq/L)	114	1.39–1.89
Total Protein (g/dL)	107	5.83–7.33
Albumin (g/dL)	112	4.50–5.56
Globulin (g/dL)	114	0.81–2.36
A/G Ratio	114	1.55–4.91
Glucose (mg/dL)	111	69.53–113.51
ALT (U/L)	113	10.19–79.95
AST (U/L)	114	81.87–480.08
Alkaline Phosphatase (U/L)	115	27.98–668.22
GGT (U/L)	115	8.08–46.35
Total Bilirubin (mg/dL)	115	0.00–0.16
Direct Bilirubin (mg/dL)	120	0.00–0.13
Indirect Bilirubin (mg/dL)	119	0.00–0.14
Amylase (U/L)	119	0.00 - <3.00
Lipase (U/L)	115	4.57–8.37
Cholesterol (mg/dL)	114	116.75–218.81
Creatine Kinase (U/L)	116	65.72–197.32
LDH (U/L)	113	265.23–557.58
Iron (ug/dL)	114	42.44–323.69
TIBC (ug/dL)	114	205.13–391.54
FE saturation (%)	120	15.98–100.00
Lipemia	119	1.12–10.60
Hemolysis	112	0.00–25.61
Icterus	120	0.00–0.00
Triglycerides (mg/dL)	110	17.69–84.40
Fibrinogen (mg/dL)	114	138.75–273.92
**Species: *Tursiops truncates***		
**Lab: UNAM**		
Hematocrit (%)	156	37.02–46.71
Hemoglobin (g/L)	153	130.59–159.34
Erythrocytes (x 10^12^/L)	157	0.00–7.44
VGM (fL)	155	108.16–146.47
CGMH (g/L)	156	322.93–369.82
Reticulocytes (x 10^9^/L)	151	0.08–2.75
Platelets (x 10^9^/L)	148	50.04–200.52
Total Solids (g/L)	155	61.62–82.78
Leukocytes (x 10^9^/L)	150	3.33–7.76
Segmented Neutrophils (x 10^9^/L)	147	1.75–4.82
Band Neutrophils (x 10^9^/L)	157	0.00–0.04
Metamyelocytes (x 10^9^/L)	157	0.00–0.00
Myelocytes (x 10^9^/L)	157	0.00–0.00
Lymphocytes (x 10^9^/L)	151	0.29–2.20
Monocytes (x 10^9^/L)	155	0.00–0.36
Eosinophiles (x 10^9^/L)	151	0.00–1.49
Basophils (x 10^9^/L)	155	0.00–0.00
RDW%	155	13.45–16.68
RDWa	155	90.43–123.20
MHC	156	32.34–55.25
MPV	150	3.19–21.75
Nucleated erythrocytes (/100 Leuc)	148	0.00–3.08
Fb (g/L)	149	1.14–2.59
Pt/Fin	80	27.87–42.88
Glucose (mmol/L)	155	4.06–6.91
Urea (mmol/L)	156	12.30–19.96
Creatinine (μmol/L)	154	100.77–177.48
Cholesterol (mmol/L)	156	3.27–7.36
Total bilirubin (μmol/L)	160	0.00–5.77
Conjugated bilirubin (μmol/L)	159	0.29–1.29
Unconjugated bilirubin (μmol/L)	158	0.00–4.57
Globulin (g/L)	155	17.46–31.57
ALT (U/L)	153	2.26–82.04
AST (U/L)	152	103.94–493.22
FA (U/L)	159	60.97–1018.50
GGT (U/L)	160	0.00–105.31
CK (U/L)	155	49.70–202.74
Total Protein (g/L)	155	57.47–75.14
Albumin (g/L)	158	36.45–46.86
A/G Ratio	156	1.18–2.24
Calcium (mmol/L)	156	1.94–2.54
Phosphorus (mmol/L)	158	0.99–1.72
Ca/P Ratio	161	1.09–2.23
Potassium (mmol/L)	162	2.78–4.00
Sodium (mmol/L)	159	147.31–158.32
Chloride (mmol/L)	159	117.73–129.19
Bicarbonate (mmol/L)	156	21.38–34.14
Anion Gap (mmol/L)	118	0.25–14.59
Strong ion Ratio (mmol/L)	158	23.34–35.66
Osmolality (mOsm/kg)	158	302.17–327.84
Triglycerides (mmol/L)	154	0.18–1.13
Iron (μmol/L)	154	3.17–56.66
Magnesium (mmol/L)	160	0.52–0.97
Amylase (U/L)	159	0.00–11.31
Lipase (U/L)	155	0.24–43.98
**Species: *Tursiops aduncus***		
**Lab: AHDC**		
Hematocrit (%)	30	38.54–50.26
Hemoglobin (g/dL)	30	13.89–17.58
RBC (mill/uL)	31	3.28–4.36
MCV (fL)	31	107.82–125.86
MCH (pg)	30	37.72–44.74
MCHC (g/dL)	29	33.61–37.01
RDW (%)	30	12.83–15.48
Reticulocyte Count (%)	26	0.99–5.32
Absolute Reticulocyte Count (thou/uL)	27	40.04–206.76
Nucleated Red Blood Cells (/100 WBC)	43	0.00–0.97
WBC (thou/uL)	30	3.12–7.36
Segmented Neutrophils (thou/uL)	46	0.00–5.22
Band Neutrophils (thou/uL)	46	0.00–0.00
Lymphocytes(thou/uL)	46	0.00–1.66
Monocytes (thou/uL)	46	0.00–0.44
Eosinophils (thou/uL)	47	0.00–1.45
Basophils (thou/uL)	45	0.00–0.00
Platelet Count (thou/uL)	30	42.82–163.64
MPV (fL)	30	12.14–20.07
TP-Ref (g/dL)	23	6.33–8.48
Sodium (mEq/L)	47	152.95–160.83
Potassium (mEq/L)	46	2.85–3.90
Chloride (mEq/L)	46	115.18–122.38
Bicarbonate (mEq/L)	46	20.93–27.07
Anion Gap (mEq/L)	47	14.56–20.71
Na/K Ratio	44	39.84–52.12
Urea Nitrogen (mg/dL)	44	34.41–59.37
Creatinine (mg/dL)	45	1.08–2.06
Calcium (mg/dL)	46	8.50–9.88
Phosphate (mg/dL)	45	3.15–5.20
Magnesium (mEq/L)	46	1.40–2.05
Total Protein (g/dL)	46	5.59–7.32
Albumin (g/dL)	47	4.11–5.19
Globulin (g/dL)	44	1.20–2.38
A/G Ratio	46	1.62–3.49
Glucose (mg/dL)	47	57.73–119.42
ALT (U/L)	47	18.43–125.03
AST (U/L)	43	102.92–444.15
Alkaline Phosphatase (U/L)	43	0.00–1444.80
GGT (U/L)	44	15.74–53.26
Total Bilirubin (mg/dL)	44	0.02–0.32
Direct Bilirubin (mg/dL)	47	0.00–0.16
Indirect Bilirubin (mg/dL)	46	0.00–0.27
Amylase (U/L)	47	0.00 - <3.00
Lipase (U/L)	45	3.14–9.66
Cholesterol (mg/dL)	46	82.13–217.43
Creatine Kinase (U/L)	45	66.27–197.20
LDH (U/L)	46	357.58–728.59
Iron (ug/dL)	45	84.85–225.20
TIBC (ug/dL)	45	215.21–489.15
FE saturation (%)	45	20.56–67.70
Lipemia	45	3.70–11.10
Hemolysis	44	0.00–36.44
Icterus	47	0.00–0.73
Triglycerides (mg/dL)	46	15.07–60.32
Fibrinogen (mg/dL)	46	150.25–307.71
**Species: *Delphinapterus leucas***		
**Lab: AHDC**		
Hematocrit (%)	21	44.63–54.32
Hemoglobin (g/dL)	19	18.13–21.40
RBC (mill/uL)	21	2.74–3.52
MCV (fL)	21	153.89–163.15
MCH (pg)	19	59.07–68.62
MCHC (g/dL)	20	37.32–42.58
RDW (%)	20	8.14–11.00
Reticulocyte Count (%)	19	0.39–1.72
Absolute Reticulocyte Count (thou/uL)	19	14.04–51.18
Nucleated Red Blood Cells (/100 WBC)	21	0.00–0.00
WBC (thou/uL)	21	4.14–8.24
Segmented Neutrophils (thou/uL)	20	2.20–5.32
Band Neutrophils (thou/uL)	20	0.00–0.09
Lymphocytes(thou/uL)	21	0.39–2.89
Monocytes (thou/uL)	20	0.00–0.60
Eosinophils (thou/uL)	21	0.00–0.62
Basophils (thou/uL)	21	0.00–0.07
Platelet Count (thou/uL)	21	81.70–168.50
MPV (fL)	20	10.16–13.62
TP-Ref (g/dL)	19	7.06–8.30
Sodium (mEq/L)	20	154.33–160.87
Potassium (mEq/L)	21	3.33–4.28
Chloride (mEq/L)	20	113.10–119.90
Bicarbonate (mEq/L)	21	22.60–30.73
Anion Gap (mEq/L)	21	14.05–21.67
Na/K Ratio	20	35.65–48.15
Urea Nitrogen (mg/dL)	20	43.66–61.34
Creatinine (mg/dL)	21	0.61–1.53
Calcium (mg/dL)	20	8.87–10.21
Phosphate (mg/dL)	21	4.48–6.77
Magnesium (mEq/L)	21	1.50–2.15
Total Protein (g/dL)	20	6.03–7.32
Albumin (g/dL)	21	3.80–5.35
Globulin (g/dL)	20	1.14–3.01
A/G Ratio	19	1.32–3.20
Glucose (mg/dL)	21	66.80–116.24
ALT (U/L)	21	2.55–18.11
AST (U/L)	19	46.70–82.77
Alkaline Phosphatase (U/L)	20	21.64–182.86
GGT (U/L)	19	7.91–22.41
Total Bilirubin (mg/dL)	21	0.00–0.07
Direct Bilirubin (mg/dL)	21	0.00–0.00
Indirect Bilirubin (mg/dL)	21	0.00–0.07
Amylase (U/L)	21	0.00–3.00
Lipase (U/L)	21	3.81–7.05
Cholesterol (mg/dL)	21	138.97–254.17
Creatine Kinase (U/L)	20	34.63–233.47
LDH (U/L)	20	155.28–317.12
Iron (ug/dL)	19	113.54–370.35
TIBC (ug/dL)	20	370.49–727.21
FE saturation (%)	20	20.30–70.10
Lipemia	20	5.43–13.87
Hemolysis	21	0.17–66.50
Icterus	21	0.00–0.00
Triglycerides (mg/dL)	20	68.16–231.84
Fibrinogen (mg/dL)	20	63.44–134.46
**Species: *Lagenorhynchus obliquidens***		
**Lab: AHDC**		
Hematocrit (%)	7	48.00–53.00
Hemoglobin (g/dL)	7	17.40–19.10
RBC (mill/uL)	7	5.00–5.90
MCV (fL)	7	88.00–97.00
MCH (pg)	7	32.00–35.00
MCHC (g/dL)	7	35.00–37.00
RDW (%)	7	12.80–13.40
Reticulocyte Count (%)	7	1.70–3.00
Absolute Reticulocyte Count (thou/uL)	7	94.00–160.00
Nucleated Red Blood Cells (/100 WBC)	7	0.00–2.00
WBC (thou/uL)	7	2.40–4.90
Segmented Neutrophils (thou/uL)	7	0.80–3.00
Band Neutrophils (thou/uL)	7	0.00–0.00
Lymphocytes(thou/uL)	7	0.50–1.10
Monocytes (thou/uL)	7	0.10–0.20
Eosinophils (thou/uL)	7	0.70–1.20
Basophils (thou/uL)	7	0.00–0.10
Platelet Count (thou/uL)	7	117.00–178.00
MPV (fL)	7	12.30–14.40
TP-Ref (g/dL)	7	6.50–7.00
Sodium (mEq/L)	7	158.00–160.00
Potassium (mEq/L)	7	3.10–3.30
Chloride (mEq/L)	7	113.00–117.00
Bicarbonate (mEq/L)	7	29.00–32.00
Anion Gap (mEq/L)	7	14.00–17.00
Na/K Ratio	7	48.00–51.00
Urea Nitrogen (mg/dL)	7	38.00–46.00
Creatinine (mg/dL)	7	0.60–0.80
Calcium (mg/dL)	7	8.30–8.90
Phosphate (mg/dL)	7	4.00–5.10
Magnesium (mEq/L)	7	1.50–1.80
Total Protein (g/dL)	7	5.60–6.50
Albumin (g/dL)	7	3.70–4.30
Globulin (g/dL)	7	1.30–2.60
A/G Ratio	7	1.50–3.30
Glucose (mg/dL)	7	87.00–114.00
ALT (U/L)	7	75.00–188.00
AST (U/L)	7	210.00–358.00
Alkaline Phosphatase (U/L)	7	166.00–397.00
GGT (U/L)	7	20.00–27.00
Total Bilirubin (mg/dL)	7	0.00–0.00
Direct Bilirubin (mg/dL)	7	0.00–0.00
Indirect Bilirubin (mg/dL)	7	0.00–0.00
Amylase (U/L)	7	<3.00 - <3.00
Lipase (U/L)	7	5.00–6.00
Cholesterol (mg/dL)	7	132.00–165.00
Creatine Kinase (U/L)	7	138.00–186.00
LDH (U/L)	7	548.00–675.00
Iron (ug/dL)	7	112.00–220.00
TIBC (ug/dL)	7	324.00–414.00
FE saturation (%)	7	32.00–71.00
Lipemia	7	3.00–6.00
Hemolysis	7	8.00–41.00
Icterus	7	0.00–0.00
Triglycerides (mg/dL)	7	34.00–80.00
Fibrinogen (mg/dL)	7	122.00–176.00

*Note*. n refers to the number of samples.

General 95% reference intervals and values (without accounting for sex, age, age^2^, and month) are given below if there were no significant predictors of the variable for common and Indo-Pacific bottlenose dolphins and beluga whales or if access to the iOS ZooPhysioTrak app is not available. Non-parametric 90% reference values are given for Pacific white-sided dolphins.

**Fig 1:**
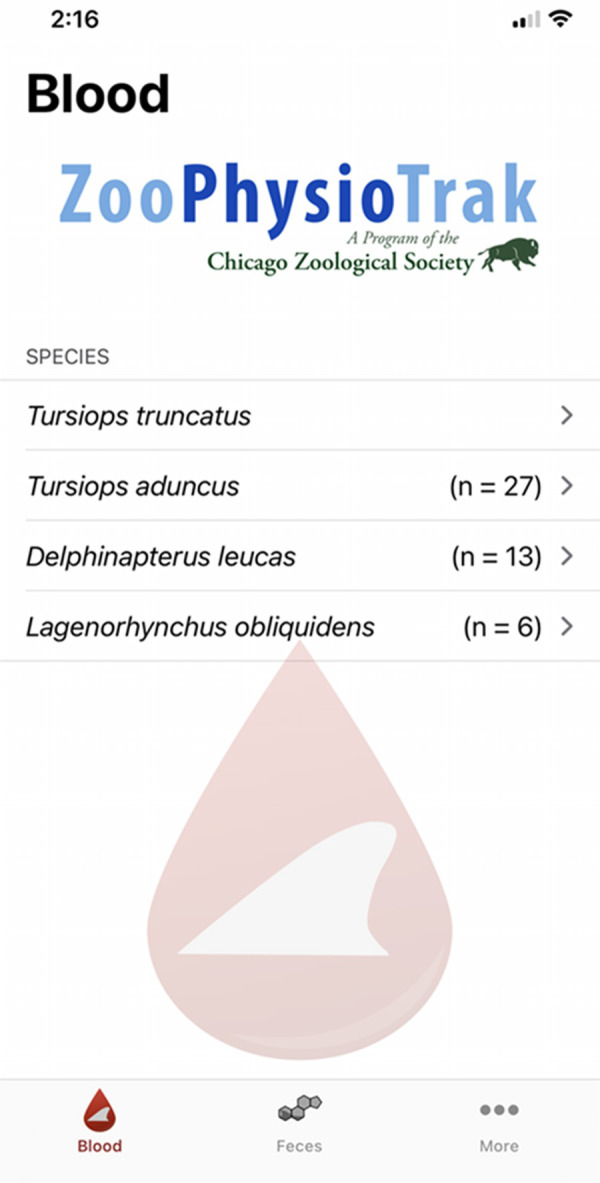
Screenshot taken on an iPhone device illustrating the user interface for the species selection screen.

**Fig 2:**
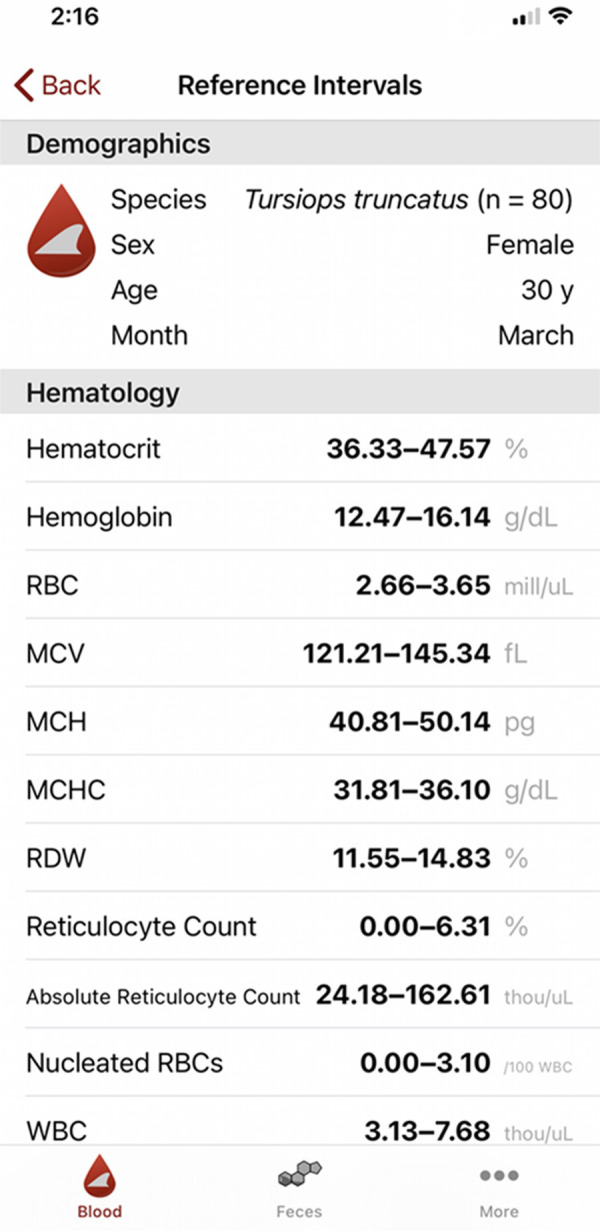
Screenshot taken on an iPhone device illustrating the user interface for the reference interval screen.

ZooPhysioTrak was designed to provide a feature that supplies hematological, serum, and plasma biochemical reference intervals and values for common bottlenose dolphins, Indo-Pacific bottlenose dolphins, beluga whales, and Pacific white-sided dolphins (Figs 1 and 2). Users input demographic variables for an individual including species, diagnostic laboratory (AHDC or UNAM for common bottlenose dolphins only), age, sex, and month. The application returns the appropriate reference intervals and values accounting for significant factors affecting each variable.

The application took a no-keyboard approach. All data is entered via switches, segmented controls, and rotating pickers. Only valid data choices are displayed. If an interval can not be calculated, a reason such as “need age” or “no data” is displayed as appropriate. ZooPhysioTrak is compatible for side-by-side work with other applications. Data is not linked to a database for exportation; however, variables are listed in the same formatting order as provided by the diagnostic laboratories for easy comparison.

## Discussion

Reference intervals and values for hematological, serum, and plasma variables established from healthy individuals have been previously reported for a variety of cetacean species living in the wild and under professional care [1, 5, 6, 9, 19–22]. Due to collection variability, diagnostic machine type, and varying levels of processing skill, values reported for blood samples analyzed at different diagnostic laboratories are not directly comparable [19, 22]. Thus, reference intervals for specific values presented in prior reports are limited in their usefulness as health assessment tools for future samples collected and were processed following different protocols. To amend this limitation, samples in the present study were processed at service laboratories that are available to analyze future samples and protocols for collecting and shipping samples are provided in the ZooPhysioTrak iOS application. Comparisons of individual values over time at a common laboratory is essential for health trend determination and most applicable at smaller facilities where animal numbers are limited.

Blood samples are tested at in-house laboratories or through a variety of commercial diagnostic laboratories. Laboratory choice is often determined by the historical use of a specific laboratory, physical proximity to aid in rapid turnaround of results for ill animals, trust of results, and economic cost including both shipping and analysis. Samples tested at different laboratories should only be compared following appropriate statistical analysis of parallel samples because the variation in results between testing laboratories leads to differences in reference intervals or values [23]. For example, the monocyte reference interval for common bottlenose dolphins in the present study was 0.00–0.47 thou/uL and was 0.00–0.57 thou/uL as previously reported by Venn-Watson, Jensen, and Ridgway [7].

The present study expands upon these reports for common bottlenose dolphins, beluga whales, and Pacific white-sided dolphins. Previously undescribed hematological, serum, and plasma reference values for Indo-Pacific bottlenose dolphins were established. Sex, age, season, and nutrition affect various clinicopathologic variables in cetaceans [2, 7, 19, 24]. Similarly, sex, age, age^2^, and month of the year were all found to be important determinants of several blood variables in the present data as well.

In wild common bottlenose dolphins, juveniles had higher TIBC concentrations than adult dolphins [20, 25]. This relationship was also apparent for Indo-Pacific bottlenose dolphins in this study. Additionally, TIBC values were significantly higher for female dolphins than male dolphins which was similar to findings from common bottlenose dolphins under professional care [26]. This may reflect ages that have a higher need for iron availability for growth and replacement after contributing iron to fetal development.

Alkaline phosphatase decreased with age for common bottlenose dolphins tested at AHDC and for Indo-Pacific bottlenose dolphins. Alkaline phosphatase is associated with the osseous activity that occurs during early growth [27, 28]. Prior studies have reported a decrease in alkaline phosphatase with age in common bottlenose dolphins under professional care [7]. Decreased alkaline phosphatase is also associated with inflammation and illness as well as decreased food intake, or to seasonal changes in the fat content of prey fish fed [29]. In addition to Alkaline phosphatase, calcium is also associated with active bone formation [30]. For beluga whales, calcium significantly decreased with age in the present study. Calcium has been reported to be higher in juvenile beluga whales, Yangtze finless porpoises, killer whales, and bottlenose dolphins [2, 20, 31, 32]. Calcium values were significantly higher for female Indo-Pacific bottlenose dolphins than male dolphins.

In Indo-Pacific bottlenose dolphins and common bottlenose dolphin samples analyzed at AHDC, hematocrit decreased with age. These trends were consistent with previous reports of hematocrit and platelet counts in common bottlenose dolphins under professional care [7]. It is important that clinicians also follow cell size, MCH, and MCHC to detect variables that can influence hematocrits determined by machine calculations. Spun PCV is still considered a valuable tool to run in tandem for quality control when decreases in HCT are detected. Consistent with reports of prior reticulocyte values of beluga whales under professional care [8], female beluga whales had higher reticulocyte counts than males. However, Norman and colleagues [8] found an increase in reticulocyte count with age which is inconsistent with the present results which indicated a decrease with age.

For common bottlenose dolphin samples analyzed at the AHDC lab and Indo-Pacific bottlenose dolphins, creatinine significantly increased with age. These findings were consistent with previous reports of creatinine values increasing with age for common bottlenose dolphins under professional care [7, 24]. Seasonality affects creatine levels in Yangtze finless porpoises, beluga whales, and common bottlenose dolphins [19, 21, 33]. It is hypothesized that the difference is a result of changes in nutritional status due to seasonal prey availability and an increase in muscle mass during the summer months [24]. Hall and colleagues [19] suggested that the changes may be more reflective of blubber thickness rather than muscle mass. This is consistent with clinical diet management and seasonal variations in fat content per kilogram of wild fish fed where high fat fish contain additional metabolic water. Month was not a significant predictor of creatinine concentrations for any of the focal species consistent with reports of belugas under professional care [9]. It is possible that no significant difference was apparent because food availability remained constant for the dolphins and whales in the present study.

In the present study, creatinine kinase significantly decreased with age in common bottlenose dolphin samples analyzed at the AHDC laboratory. Juvenile common bottlenose dolphins in other populations also had significantly higher creatinine kinase than adults [7, 20, 25–26]. Similar trends were reported for Yangtze finless porpoises and beluga whales [9, 33]. When compared to male dolphins, lymphocyte values counts were significantly higher in female common bottlenose dolphin samples analyzed at the AHDC laboratory. Lymphocyte count also significantly decreased with age. This is consistent with findings from female common bottlenose dolphins [7] and Yangtze finless porpoises [33].

Significant predictors for blood variables were different for the two groups of common bottlenose dolphin samples that were tested at different diagnostic laboratories. It is possible that these differences were due to inter-laboratory variation. Another potential reason for the difference in predictors is the habitat locations. The majority of samples sent to the UNAM lab were obtained from animals housed in professionally managed ocean habitats while the majority of samples sent to the AHDC lab were housed in professionally managed zoo/aquarium habitats. Seasonal changes affect many hematological and serum biochemical values of wild dolphins [19, 24]. Blubber thickness increases in response to the decrease in water temperature and increased fatty fish available during winter months [34]. It is possible that month was a more common predictor for the samples analyzed at the UNAM lab due to the changes in ocean temperature experienced by the dolphins or as a result of dietary or management differences.

Changes in triglyceride values are associated with dietary changes, lipid metabolism, and potentially hormonal factors. Consistent with findings in wild common bottlenose dolphins [20], triglyceride values were higher for female common bottlenose dolphin samples than male dolphins tested at UNAM. Triglyceride values for female Indo-Pacific bottlenose dolphins were also significantly higher than male dolphins. Triglyceride values for common bottlenose dolphins samples tested at ADHC significantly increased with age similar to prior reports of values from common bottlenose dolphins under professional care [7]. However, triglycerides have also been shown to decrease with increased age in wild common bottlenose dolphins [20].

In common bottlenose dolphins, juveniles have higher mean concentrations of blood urea nitrogen than adults [20, 25]. In contrast, Indo-Pacific bottlenose dolphin urea nitrogen concentrations significantly increased with age and was also significantly affected by month with lower values reported later in the year. Consistent with findings from common bottlenose dolphins [24, 25], cholesterol was significantly higher for female Indo-Pacific bottlenose dolphins than male dolphins. These differences may be a result of sex-related differences in the lipid profile [35], or changes in diet management and seasonal variation that normally occurs in prey species. In the present study, fibrinogen values were found to be significantly higher in female Indo-Pacific bottlenose dolphins than male dolphins. This relationship has previously been reported for pregnant female common bottlenose dolphins [20].

The ZooPhysioTrak iOS application was developed in order to facilitate easy access to the reference intervals and values for the four species of cetacean. The application supplies a streamlined method of viewing reference intervals and values for an individual while taking into account significant predictors. ZooPhysioTrak delivers valuable information that can be used by animal care professionals to guide health assessments and decisions.

This work allows the cetacean facility clinician and care providers to see the comparative health results of quality controlled, commonly used diagnostic tests usually run in many unrelated laboratories. Laboratory choice is often determined by the need for a rapid turnaround time with ill animals, trust of results, and economic considerations that include test costs and mailing expense. Data quality can be affected by machine type, technicians with varying levels of experience and skill, interpretation of cytology findings, and variations in quality control. This presents one limitation of the present study. While the samples were analyzed following standardized protocols at both testing laboratories, it is possible that there is intra-testing variability within the same lab because the samples were not tested as a single batch. However, this variability would fall within the range that would occur when using these values to compare results from future samples.

Following current best practice, reference intervals and values from laboratories that are generated from healthy conspecifics can be used to complement robust individual reference intervals. Generally, voluntary blood samples can be frequently collected from cetaceans and used to build a database of an individual’s historic reference values. Both species and individual level reference intervals can be used in concert to inform health management decisions. Where questions of results develop for individuals, laboratories may provide a comparative bench mark with individual reference values alongside intermittent parallel samples for quality control. The list of standard health tests should be analyzed by reputable, experienced laboratories with the goal of setting in-house species and individual reference intervals over time. Importantly, a robust historical record of an individual’s blood values will allow professionals to take into account any potential variation that may exist as a result of the visitor high and low seasons. The reference ranges presented in ZooPhysioTrak include values from blood samples collected in one high and one low visitor season at each facility. The high and low visitor season varied in the month of the year that they occurred based on the geographic location. Future research should also examine if the high/low visitor seasons affect blood values.

While the reference intervals and values presented here and in ZooPhysioTrak are for healthy individuals, sustained values in the higher or lower portions of the given ranges should be closely monitored. For example, serum iron may vary depending on age, gender, environment, diet, and nutritional status [36]. Generally, iron levels greater than 350 ug/dL should be monitored on a regular basis in conjunction with TIBC and FE saturation. TIBC ranges may also vary but as seen here they are generally similar to those reported by Mazzaro and colleagues [36] where managed animals ranged from 239 to 604 ug/dL. These values were generally lower when compared to two wild dolphin populations with values running less than 400 ug/dL. Iron or FE saturation of 20% to 67.7% from the present study compare with the values reported by Mazzaro and colleagues [36] for common bottlenose dolphins under professional care ranging from 40% to 80%, while the two wild dolphin populations ranged from 22% to 68%. High levels of the three indices may be associated with the potential or presence of iron overload. Clinical iron overload is also associated with elevated liver enzymes. Thus, reference intervals and values should be used alongside in-house reference intervals to examine test results holistically and over time.

Continued research is necessary to understand the effects of diet, seasonality, and water temperature on blood values. Diet has been suggested to affect several blood variables [8, 9, 36]. Diet was not included as a variable in the present study due to the large variety of fish and cephalopod species that are fed and the various quantities in which they were fed to the participants. Additionally, dietary supplementation of water and gelatin was not accounted for. Future experimental studies related to the effects of diet and dietary supplementation of water would be beneficial. The present study utilized two samples taken approximately six months apart which represented nine months of the year across all participants. Participants experienced varying water temperature ranges based on the habitat type and/or geographic location. Some participants resided in habitats with stable year round water temperatures and others experienced seasonal temperature changes at varying magnitudes. While these data revealed insight into potential seasonal patterns, future research should investigate the impact of season and water temperatures on blood values using a denser sampling schedule over multiple years.

The hematological, serum, and plasma reference intervals and values provide an important diagnostic tool for future health assessments for cetaceans in accredited zoos and aquariums. The data are representative of cetaceans in a variety of habitats and management experiences. Sex, age, age^2^, and month predictor variables were found to be important determinants of several blood variables in the present data. For common bottlenose dolphins, the results reported here were generated from apparently healthy dolphins residing in a large number of accredited zoos and aquariums resulting in the first reported reference intervals that are applicable across facilities. This is the first report of a hematological, serum, and plasma values and reference values for Indo-Pacific bottlenose dolphins. While blood variables for beluga whales have been previously reported, the sample collection and processing protocols given in ZooPhysioTrak and reported values provide a standardized starting point for comparing future values. The reference values provided for Pacific white-sided dolphins presented an updated and expanded suite of blood parameters. The reference intervals and values and ZooPhysioTrak supply veterinarians and animal care professionals with valuable information that can be utilized in providing cetaceans in zoos and aquariums optimal care. Future research should examine the effects that other variables (*e*.*g*., habitat type, visitor attendance, and diet) may have on blood values.

## Supporting information

S1 Data Descriptive statistics lauderdale health. (XLSX)

S1 Fig Striking image lauderdale health. (TIFF)

S1 Table Lauderdale health. List of blood variables included in the analysis from the Cornell University Animal Health Diagnostic Center.
(DOCX)

S2 Table Lauderdale health. List of blood variables included in the analysis from the Universidad Nacional Autónoma de México.
(DOCX)

## Abbreviations

A/G: Ratio Albumin globulin ratio
ALT: Alanine aminotransferase
AST: Aspartate aminotransferase
Ca/P: Ratio Calcium/phosphorous ratio
CGMH: Mean corpuscular hemoglobin concentration
CK: Creatinine kinase
GGT γ: Glutamyltransferase
Fb: Fibrinogen
FA: Alkaline phosphatase
FE: Saturation Iron saturation
LDH: Lactate dehydrogenase
MCV: Mean corpuscular volume
MCH: Mean corpuscular hemoglobin
MCHC: Mean corpuscular hemoglobin concentration
MPV: Mean platelet volume
Na/K: Ratio Sodium to potassium ratio
Pt/Fin: Total protein to fibrinogen ratio
RBC: Red blood cell count
RDW: Red cell distribution width
RDWa: Red cell distribution width
TP: Ref Total protein
TIBC: Total iron-binding capacity
VGM: Mean corpuscular volume
WBC: White blood cell count
AHDC: Cornell University Animal Health Diagnostic Center
UNAM: Universidad Nacional Autónoma de México
