# Supplementary figures and images for: Health reference intervals and values for common bottlenose dolphins (Tursiops truncatus), Indo-Pacific bottlenose dolphins (Tursiops aduncus), Pacific white-sided dolphins (Lagenorhynchus obliquidens), and beluga whales (Delphinapterus leucas)
Source: PLoS One. 2021 Aug 30;16(8):e0250332. doi: 10.1371/journal.pone.0250332 (PMC8405036; doi:10.1371/journal.pone.0250332)

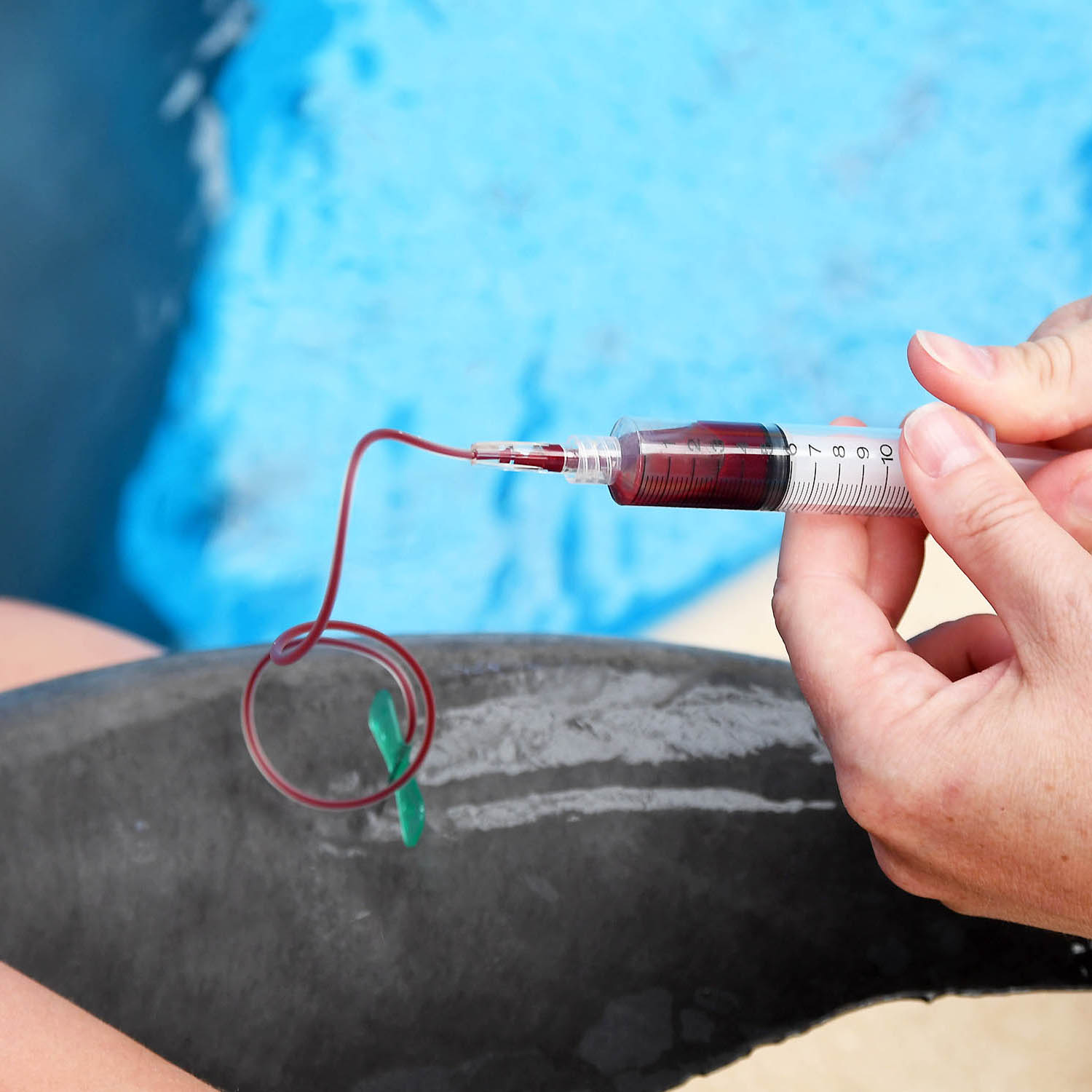

Supplement: S1 Fig — (TIFF) [file pone.0250332.s002.tiff]
