# Supplementary material for: Health reference intervals and values for common bottlenose dolphins (Tursiops truncatus), Indo-Pacific bottlenose dolphins (Tursiops aduncus), Pacific white-sided dolphins (Lagenorhynchus obliquidens), and beluga whales (Delphinapterus leucas)
Source: PLoS One. 2021 Aug 30;16(8):e0250332. doi: 10.1371/journal.pone.0250332 (PMC8405036; doi:10.1371/journal.pone.0250332)
Supplement: S1 Table — List of blood variables included in the analysis from the Cornell University Animal Health Diagnostic Center. (DOCX) [file pone.0250332.s003.docx]

**S1 TABLE**

**S1 Table. List of blood variables included in the analysis from the Cornell University Animal Health Diagnostic Center.**

| **Test** | **Variable** |  |
| --- | --- | --- |
| Hematology | Hematocrit |  |
| Hematology | Hemoglobin |  |
| Hematology | RBC |  |
| Hematology | MCV |  |
| Hematology | MCH |  |
| Hematology | MCHC |  |
| Hematology | RDW |  |
| Hematology | Reticulocyte Count |  |
| Hematology | Absolute Reticulocyte Count |  |
| Hematology | Nucleated RBCs |  |
| Hematology | WBC |  |
| Hematology | Segmented Neutrophils |  |
| Hematology | Band Neutrophils |  |
| Hematology | Lymphocytes |  |
| Hematology | Monocytes |  |
| Hematology | Eosinophils |  |
| Hematology | Basophils |  |
| Hematology | Platelet Count |  |
| Hematology | MPV |  |
| Serum Chemistry | TP-Ref |  |
| Serum Chemistry | Sodium |  |
| Serum Chemistry | Potassium |  |
| Serum Chemistry | Chloride |  |
| Serum Chemistry | Bicarbonate |  |
| Serum Chemistry | Anion Gap |  |
| Serum Chemistry | NaK Ratio |  |
| Serum Chemistry | Urea Nitrogen |  |
| Serum Chemistry | Creatinine |  |
| Serum Chemistry | Calcium |  |
| Serum Chemistry | Phosphate |  |
| Serum Chemistry | Magnesium |  |
| Serum Chemistry | Total Protein |  |
| Serum Chemistry | Albumin |  |
| Serum Chemistry | Globulin |  |
| Serum Chemistry | AG Ratio |  |
| Serum Chemistry | Glucose |  |
| Serum Chemistry | ALT |  |
| Serum Chemistry | AST |  |
| Serum Chemistry | Alkaline Phosphatase |  |
| Serum Chemistry | GGT |  |
| Serum Chemistry | Total Bilirubin |  |
| Serum Chemistry | Direct Bilirubin |  |
| Serum Chemistry | Indirect Bilirubin |  |
| Serum Chemistry | Amylase |  |
| Serum Chemistry | Lipase |  |
| Serum Chemistry | Cholesterol |  |
| Serum Chemistry | Creatine Kinase |  |
| Serum Chemistry | LDH |  |
| Serum Chemistry | Iron |  |
| Serum Chemistry | TIBC |  |
| Serum Chemistry | FE saturation |  |
| Serum Chemistry | Lipemia |  |
| Serum Chemistry | Hemolysis |  |
| Serum Chemistry | Icterus |  |
| Serum Chemistry | Triglycerides |  |
| Plasma | Fibrinogen |  |
