# Supplementary material for: Health reference intervals and values for common bottlenose dolphins (Tursiops truncatus), Indo-Pacific bottlenose dolphins (Tursiops aduncus), Pacific white-sided dolphins (Lagenorhynchus obliquidens), and beluga whales (Delphinapterus leucas)
Source: PLoS One. 2021 Aug 30;16(8):e0250332. doi: 10.1371/journal.pone.0250332 (PMC8405036; doi:10.1371/journal.pone.0250332)
Supplement: S2 Table — List of blood variables included in the analysis from the Universidad Nacional Autónoma de México. (DOCX) [file pone.0250332.s004.docx]

**S2 TABLE**

**S2 Table. List of blood variables included in the analysis from the Universidad Nacional Autónoma de México.**

| **Test** | **Variable** |
| --- | --- |
| Hemograma | Hematocrito |
| Hemograma | Hemaglobina |
| Hemograma | Eritrocitos |
| Hemograma | VGM |
| Hemograma | CGMH |
| Hemograma | Reticulocitos |
| Hemograma | Plaquetas |
| Hemograma | Solidos Totales |
| Hemograma | Leucocitos |
| Hemograma | Neutrofilos |
| Hemograma | Bandas |
| Hemograma | Metamielocitos |
| Hemograma | Mielocitos |
| Hemograma | Linfocitos |
| Hemograma | Monocitos |
| Hemograma | Eosinofilos |
| Hemograma | Basofilos |
| Hemograma | RDW% |
| Hemograma | RDWa |
| Hemograma | MHC |
| Hemograma | MPV |
| Hemograma | Eritrocitos Nucleados |
| Hemograma | Fb |
| Hemograma | Pt/Fin |
| Bioquímica | Glucosa |
| Bioquímica | Urea |
| Bioquímica | Creatinina |
| Bioquímica | Colesterol |
| Bioquímica | Bilirrubina Total |
| Bioquímica | Bilirrubina Conjugada |
| Bioquímica | Bilirrubina No Conjugada |
| Bioquímica | Gobulinas |
| Bioquímica | Alanina Aminotransferase (ALT) |
| Bioquímica | Aspartato Aminotransferase (AST) |
| Bioquímica | Fosfatasa Alcalina (FA) |
| Bioquímica | Gamaglutamil Transferasa (GGT) |
| Bioquímica | Creatin Quinasa (CK) |
| Bioquímica | Proteinas Totales |
| Bioquímica | Alnumina |
| Bioquímica | Relacion A/G |
| Bioquímica | Calcio |
| Bioquímica | Fosforo |
| Bioquímica | Relacion Ca/P |
| Bioquímica | Potasio |
| Bioquímica | Sodio |
| Bioquímica | Cloro |
| Bioquímica | Bicarbonato |
| Bioquímica | Anion Gap |
| Bioquímica | Diferencia de Iones Fuertes |
| Bioquímica | Osmolalidad |
| Bioquímica | Trigliceridos |
| Bioquímica | Hierro |
| Bioquímica | Magnesio |
| Bioquímica | Amilasa |
| Bioquímica | Lipasa |
